# Associations among maternal pre-pregnancy body mass index, gestational weight gain and risk of autism in the Han Chinese population

**DOI:** 10.1186/s12888-018-1593-2

**Published:** 2018-01-17

**Authors:** Yidong Shen, Huixi Dong, Xiaozi Lu, Nan Lian, Guanglei Xun, Lijuan Shi, Lu Xiao, Jingping Zhao, Jianjun Ou

**Affiliations:** 1 Institute of Mental Health, The Second Xiangya Hospital, Central South University; National Clinical Research Center on Mental Disorders, The National Technology Institute on Mental disorders; Hunan Key Laboratory of Psychiatry and Mental Health, Changsha, China; 2 Qingdao Mental Health Center, Qingdao, China; 3 The First Affiliated Hospital of Zhengzhou University, Zhengzhou, China; 4 Mental Health Center of Shandong Province, Jinan, China; 5 Neuropsychology and Applied Cognitive Neuroscience Research Group, Institute of Psychology, Chinese Academy of Sciences, Beijing, China

**Keywords:** Autism, Gestational weight gain, Body mass index, Pregnancy

## Abstract

**Background:**

Autism is a neurodevelopmental disorder with an unclear etiology. Pre-pregnancy body mass index (BMI) and gestational weight gain (GWG) have been suggested to play a role in the etiology of autism. The current study explores the associations among maternal pre-pregnancy BMI, GWG and the risk of autism in the Han Chinese population.

**Methods:**

Demographic information, a basic medical history and information regarding maternal pre-pregnancy and pregnancy conditions were collected from the parents of 705 Han Chinese children with autism and 2236 unrelated typically developing children. Binary logistic regressions were conducted to calculate the odds ratio (OR) for the relationship among pre-pregnancy BMI, GWG and the occurrence of autism. The interaction between pre-pregnancy BMI and GWG was analyzed by performing stratification analyses using a logistic model.

**Results:**

After adjusting for the children’s gender, parental age and family annual income, excessive GWG was associated with autism risk in the entire sample (OR = 1.327, 95% CI: 1.021–1.725), whereas the relationship between maternal pre-pregnancy BMI and autism was not significant. According to the stratification analyses, excessive GWG increased the risk of autism in overweight/obese mothers (OR = 2.468, 95% CI: 1.102–5.526) but not in underweight or normal weight mothers.

**Conclusions:**

The maternal pre-pregnancy BMI might not be independently associated with autism risk. However, excessive GWG might increase the autism risk of offspring of overweight and obese mothers.

**Electronic supplementary material:**

The online version of this article (10.1186/s12888-018-1593-2) contains supplementary material, which is available to authorized users.

## Background

Autism is a neurodevelopmental disorder characterized by impairments in social interactions and communication skills and stereotyped behaviors and interests [1]. According to the most recent epidemiological study, the prevalence of Autism Spectrum Disorder (ASD is 1.46% (1 per 68 persons) in the US [2]. The increasing prevalence of autism calls for further investigations of its unclear etiology [3].

An aberrant maternal pre-pregnancy body mass index (BMI) and gestational weight gain (GWG) have been suggested to increase the autism risk among offspring [4–7]. Although the relevant mechanism is not fully understood, maternal nutritional balance has been shown to be important to the fetus and directly influence fetal neurodevelopment [8]. Additionally, maternal metabolic status may also exert indirect influences on neurodevelopment via the immune or endocrine system [9, 10]. Thus, maternal metabolism before and during pregnancy may be involved in the etiology of autism.

In addition to the unclear mechanism, many other questions remain unanswered regarding the associations between autism and BMI/GWG. For example, although several studies enrolled a large number of participants, the number of studies examining this association is limited, and the findings were not always consistent, indicating that more replication studies are needed. Furthermore, in some US population- based cohort studies, clear ethnic differences in BMI and GWG have been observed in individuals with different genetic backgrounds, living environments and lifestyles [11, 12]. Similarly, different classes of BMI might be related to GWG values that vary by ethnicity [13]. Hence, replicated and verified the associations of autism risk with BMI and GWG in different ethnic populations are necessary. To the best of our knowledge, few studies have focused on this issue in the Han Chinese population. This study explored the possible associations among maternal pre-pregnancy BMI, GWG and the risk of autism in the Han Chinese population.

## Methods

### Subjects

This study was a part of a larger research project that was approved by the Human Ethics Committee of the Second Xiangya Hospital of Central South University.

Study subjects in the autism group were recruited by the Outpatient Department of the Mental Health Institute at the Second Xiangya Hospital of Central South University, Changsha, Hunan Province and at Elim Training Center for Children with Autism, Qingdao, Shandong Province. After obtaining written informed consent from the children’s parents or legal guardians, the children, who were aged 2–9 years, were independently diagnosed by two senior psychiatric doctors according to the DSM-IV-TR criteria for autistic disorder. Children with organic diseases of the nervous system or other psychiatric disorders, such as childhood schizophrenia, were excluded. Patients with a chromosomal abnormality were also excluded. Children with autism whose mothers experienced preterm labor (less than 37 gestational weeks) or multiple pregnancies were excluded from our study because these mothers’ GWG values may differ from those of mothers with a single fetus and a full-term birth.

In the control group, typically developing (TD) children within the same age range were recruited by adapting a network survey questionnaire based on the most popular real-name social networking platforms in China mainland. The exclusion criteria were the same as the criteria used for the autism group. Additionally, the children were excluded if their parents reported a suspected developmental problem. In total, 2499 children who had never been diagnosed with a psychiatric or developmental disorder and did not have siblings with autism were recruited; the parents of 119 (4.8%) recruited children expressed concerns regarding their child’s development. In addition, 154 (6.2%) children were preterm or multiple births and were excluded from the study based on the exclusion criteria.

Ultimately, 705 children with autism and 2236 unrelated TD children were enrolled for the subsequent data analyses. All subjects were Han Chinese.

### Data collection

The parents of the children enrolled in the autism group were asked to complete a self-administered structured questionnaire that included family demographic information, basic medical history and information regarding the maternal pre-pregnancy and pregnancy conditions.

The parents of the children in the control group were solicited through a link to our website. The front page of the website contained a brief explanation of the study. If the parents agreed to participate in the study, they were asked to complete an online questionnaire that contained the same information as the paper questionnaire and included the parents’ general impression of their child’s development.

### BMI and GWG

Pre-pregnancy BMI was calculated as weight (kg) divided by height (m) squared and was categorized into the following four groups according to the adult BMI classification standards for the Chinese population [14]: lean (<18.5 kg/m^2^), normal (18.5 kg/m^2^ to 24.0 kg/m^2^), overweight (24.0 kg/m^2^ to 28.0 kg/m^2^) and obese (≥28.0 kg/m^2^).

The Institute of Medicine (IOM) recommendation for GWG may not be suitable for Chinese women, and no official GWG recommendation is available in China. However, several studies have attempted to provide a Chinese GWG recommendation. In the current study, we adopted the criteria used by Yang [15], who calculated the quartile values from data obtained from a large general population (85,729 pregnant women and their children) and defined adequate GWG as follows: 15–22 kg for lean individuals, 13–21 kg for normal individuals, 10–18 kg for overweight individuals and 9.5–17.0 kg for obese individuals. Values lower than this range were defined as inadequate GWG, and values above this range were defined as excessive GWG.

### Other covariates

Several variables, including the child’s gender, child age (continuous variable),parental age (continuous variable), maternal history of alcoholism/drug use during pregnancy (coded as yes or no) and family annual income (coded as low, medium and high; the classification standard is shown in Table 1), were chosen as potential confounders.

**Table 1:** Comparisons of the characteristics of the enrolled children and mothers in the case and control groups

	Control	Case	t/χ^2^	df	p
	(*N* = 2200)	(*N* = 697)			
1. Child age (years), mean (SD)	5.78 (2.36)	5.04 (1.08)	11.457	2552^b^	<0.001
2. Paternal age (years), mean (SD)	29.39 (4.48)	30.33 (4.30)	−4.877	2895	<0.001
3. Maternal age (years), mean (SD)	27.06 (3.67)	27.94 (3.37)	−5.890	1262^b^	<0.001
4. Birth weight (kg), mean (SD)	3.35 (0.46)	3.38 (0.45)	−1.527	2895	0.120
5. Child Sex, n (%)					
Male	1187 (54%)	599 (85.9%)	229.037	1	0.001
Female	1013 (46%)	98 (14.1%)			
6. Family annual income (Yuan), n (%)					
low <50,000	347 (15.8%)	296 (42.5%)	274.318	2	<0.001
medium 50,000–100,000	715 (32.5%)	246 (35.3%)			
high >100,000	1138 (51.7%)	155 (22.2%)			
7. Maternal history of daily alcohol use, n (%)					
Yes	9 (0.41%)	4 (0.57%)	0.322	1	0.571
No	2191 (99.59%)	693 (99.43%)			
8. Maternal history of drug use, n (%)^a^					
Yes	0	3 (0.43%)	–	–	0.014
No	2200(100%)	694 (99.57%)			

^a^Fisher’s exact test was used

^b^The standard variance was not equal, approximate t-test was adapted and adjusted the df

### Statistical analysis

Statistical analyses were conducted using IBM SPSS Statistics, Version 22.0 (Armonk, NY). Prior to the analysis, we used box-plots (case and control groups were analyzed separately) to identify and remove the outliers in the maternal pre-pregnancy BMI and GWG values, and 4 children with autism and 26 TD children were removed from the subsequent analyses. Additionally, four children with autism and 10 TD children were removed from further analysis because their mothers reported losing weight during pregnancy. These children were removed because of their small proportion, and weight lost during pregnancy may be caused by problems or diseases.

Student’s t-test and a Chi-square test were performed to compare the demographic characteristics. Student’s t-test was also performed to analyze the differences in BMI and GWG between the case and control groups. Binary logistic regressions were conducted to calculate the odds ratio (OR) for the relationships among pre-pregnancy BMI, GWG and the occurrence of autism in the entire sample. We conducted a stratification analysis and divided the samples into subgroups based on the BMI categories, and a logistic regression was performed to explore the relationship between GWG and autism in different BMI-level samples. The selected potential confounders were entered into the regression function as covariates. The significance level of all tests was *p* < 0.05 (two-tailed).

### Sensitivity analysis

Because the data were mostly based on the parents’ self-reports, we conducted a sensitivity analysis to address the potential misclassification of GWG and BMI. We adapted the World Health Organization (WHO) BMI classifications [16] and IOM GWG recommendation [17] as different standards in the sensitivity analysis (Additional file 1: Table S1) and then repeated the stratification analysis. The range of the WHO BMI standards for underweight was the same but that for the other classifications was wider than that of the Chinese standards. The range of the IOM recommendations for adequate weight gain was narrower and that for excessive weight gain was wider than that of the Chinese recommendation.

## Results

### Sample characteristic comparisons

Comparisons of the characteristics of the sample revealed a minor but statistically significant difference in the mean age between the children with autism and the TD children (5.78 years, SD = 2.36 years in the TD children vs. 5.04 years, SD = 1.08 years in the children with autism, *t* = 11.457, df = 2552, *p* < 0.001). Significant differences in gender (χ^2^ = 229.04, p < 0.001), annual family income (χ^2^ = 274.32, p < 0.001), paternal age (*t* = −4.877, df = 2895, p < 0.001) and maternal age (*t* = −5.890, df = 1262, p < 0.001) were also observed between the two groups. No significant difference was observed in the children’s birth weight (*t* = −1.527, df = 2895, *p* = 0.127). The detailed characteristics of the sample are shown in Table 1.

Regarding the maternal history of alcoholism/drug use during pregnancy, only 9 (0.41%) mothers in the TD group reported a history of daily alcohol use during pregnancy, and no mothers reported a history of drug use. In the autism group, 4 (0.57%) mothers reported a history of daily alcohol use, and 3 (0.43%) mothers reported a history of drug use but did not report the specific names of the drugs used. Because the proportions of mothers with a history of alcoholism/drug use during pregnancy were very low in both groups, we did not enter these two confounders into the subsequent logistic analyses.

### Maternal pre-pregnancy BMI and GWG

The comparisons of maternal pre-pregnancy BMI and GWG are shown in Table 2. The mean BMI of the mothers with TD children was 20.30 kg/m^2^ (SD = 2.27 kg/m^2^), and the mean BMI of the mothers with children with autism was 20.50 kg/m^2^ (SD = 2.25 kg/m^2^). In the TD group, 21.2% of the mothers were underweight, 71.8% of the mothers were normal weight, 6.4% of the mothers were overweight, and 0.6% of the mothers were obese according to the Chinese criteria. In the autism group, 17.5% of the mothers with children with autism were underweight, 75.0% of the mothers were normal weight, 7.0% of the mothers were overweight, and 0.5% of the mothers were obese before pregnancy. A significant group difference was observed in maternal pre-pregnancy BMI (*t* = −2.015, df = 2895, *p* = 0.044) but not in the BMI categories (χ^2^ = 4.841, *p* = 0.184).

**Table 2:** Comparisons of maternal pre-pregnancy BMI and GWG between the case and control groups

	Control	Case	Value	df	p
	(*n* = 2200)	(*n* = 697)			
1. BMI (kg/m^2^), mean (SD)	20.30 (2.27)	20.50 (2.25)	−2.015	2895	0.044
2. BMI categories, n (%)					
Underweight	466 (21.2%)	122 (17.5%)	4.841	3	0.184
Normal	1580 (71.8%)	523 (75.0%)			
Overweight	141 (6.4%)	49 (7.0%)			
Obese	13 (0.6%)	3 (0.5%)			
3. GWG (kg), mean (SD)	15.04(5.81)	15.70 (6.21)	−2.526	2895	0.012
4. GWG categories, n (%)					
Inadequate	793 (36.0%)	226 (32.4%)	7.912	2	0.019
Adequate	344 (15.6%)	139 (19.9%)			
Excessive	1063 (48.4%)	332 (47.7%)			

*BMI* body mass index, *GWG* gestation weight gain

The mean GWG in the mothers with TD children was 15.04 kg (SD = 5.81 kg), and that in the mothers with children with autism was 15.70 kg (SD = 6.21 kg). In the TD group, 36% of the mothers had an inadequate GWG, 48.3% of the mothers had an adequate GWG and 15.6% of the mothers had an excessive GWG. Among the mothers of children with autism, 32.4% had an inadequate GWG, 47.6% had an adequate GWG, and 19.9% had an excessive GWG. The analyses revealed significant differences in the GWG values (*t* = −2.526, df = 2895, *p* = 0.012) and GWG categories (χ^2^ = 7.912, *p* = 0.019).

### BMI, GWG and autism risk

A logistic regression was conducted to explore the relationship between pre-pregnancy BMI and autism risk (Table 3). The normal weight class was used as a reference. The obese group was combined with the overweight group to create an overweight/obese group because the number of subjects in this group was limited. The relationship was not significant according to the logistic model (underweight group: OR = 0.958, 95% CI: 0.746–1.231; overweight/obese group: OR = 0.743, 95% CI: 0.515–1.073).

**Table 3:** Odds ratios and 95% confidence intervals for the association among maternal pre-pregnancy BMI, GWG and autism in all subjects

	OR	95% CI	p
Maternal pre-pregnancy BMI^a^			
Underweight	0.958	0.746–1.231	0.738
Overweight/Obese	0.743	0.515–1.073	0.113
Gestational weight gain^b^			
Inadequate weight gain	0.855	0.690–1.060	0.154
Excessive weight gain	1.327	1.021–1.725	0.034
BMI by GWG	1.064	1.009–1.123	0.023

*BMI* body mass index, *GWG* gestation weight gain

Child’s gender, child age, parental age, and family annual income were used as covariates

^a^Normal BMI group was used as a reference

^b^Adequate weight gain group was used as a reference

Another logistic regression was conducted to explore the relationship between GWG and autism risk (Table 3). The adequate weight gain group was used as a reference. Excessive weight gain during pregnancy was associated with the risk of autism in the entire sample (OR = 1.327, 95% CI: 1.021–1.725). However, an inadequate gestational weight gain was not significantly associated with the risk of autism (OR = 0.855, 95% CI: 0.690–1.060). The interaction term (BMI × GWG) was also explored by introducing into a logistic equation. The result showed that the interaction effect was significantly associated with autism risk (OR = 1.064, 95% CI: 1.009–1.123).

Then we performed the stratification analyses, logistic regressions were conducted to explore the relationship between GWG and autism risk in each BMI category subgroup (the obese group was combined with the overweight group; details provided in Table 4). In the lean mothers, inadequate (OR = 1.164, 95% CI: 0.726–1.866) and excessive GWG (OR = 1.016, 95% CI: 0.502–2.055) were not significantly associated with the risk of autism. In the normal pre-pregnancy weight mothers, excessive GWG (OR = 1.289, 95% CI: 0.947–1.754) and inadequate GWG (OR = 0.784, 95% CI: 0.609–1.010) were not significantly associated with the risk of autism. In the overweight/obese mothers, excessive GWG significantly and more obviously increases the risk of autism (OR = 2.468, 95% CI: 1.102–5.526). In contrast, inadequate GWG was not significantly associated with the risk of autism in the same group (OR = 0.548, 95% CI: 0.193–1.559).

**Table 4:** Odds ratios and 95% confidence intervals for the association between GWG and autism in different maternal pre-pregnancy BMI categories

	OR	95% CI	p
Underweight^a^			
Inadequate weight gain	1.164	0.726–1.866	0.530
Excessive weight gain	1.016	0.502–2.055	0.965
Normal^a^			
Inadequate weight gain	0.784	0.609–1.010	0.059
Excessive weight gain	1.289	0.947–1.754	0.107
Overweight/Obese^a^			
Inadequate weight gain	0.548	0.193–1.559	0.260
Excessive weight gain	2.468	1.102–5.526	0.028

*BMI* body mass index, *GWG* gestation weight gain

Child’s gender, child age, parental age, and family annual income were used as covariates

^a^Adequate weight gain group was used as a reference

### Sensitivity analysis

The results of the sensitivity analyses are summarized in Additional file 1: Table S2. Adopting a different GWG and BMI standard did not alter the significance or direction of the outcomes.

## Discussion

To the best of our knowledge, this study included the largest sample to date to explore the relationship among pre-pregnancy BMI, GWG and autism in the Chinese population. In the present study, we found that being underweight or overweight before pregnancy might not be associated with the risk of autism in offspring. Many other studies have reported similar results in underweight and overweight mothers [4, 18, 19]. Bilder et al. did not identify associations between ASD risk and maternal BMI at the start of pregnancy in a population-based ASD cohort that was compared with age- and gender- matched controls [4]. In a recent published study, Jo H et al. observed an association between the risk of autism/developmental delay and obese class II/III (BMI ≥ 35) mothers but not with underweight or overweight mothers in a general population-based, prospective study (although the pre-pregnancy BMI data were retrospectively collected, as the subjects were enrolled during the third trimester of pregnancy) [18]. Suren P et al. conducted a similar population-based, prospective study involving a Norwegian cohort and reported that only maternal obesity (BMI ≥ 30), but not underweight or overweight, was weakly associated with the risk of ASD (adjusted OR = 1.34, 95% CI: 0.84–2.12) [19]. However, other studies have reported different results [6, 7]. In a population-based, case-control study, Getz K et al. reported a significant U-shaped association between maternal BMI and ASD occurrence, and mothers who were underweight before pregnancy were associated with a significantly increased risk of their offspring developing ASD (OR = 1.43 95% CI: 1.01, 2.04) [7]. In a most recent study involving a Danish national birth cohort consisting of 81,892 mother-child pairs, Andersen CH et al. also reported a U-shaped association between pre-pregnancy BMI and ASD [20]. We suspect that the differences in the socio-demographic characteristics and genetic backgrounds might account for the inconsistency in the results among the studies. For example, recently, Gardner et al. [6] reported that the significant association between maternal BMI and the risk of ASD among offspring disappeared when cases were compared to their unaffected siblings. Several other important but ignored confounders, such as paternal obesity, could be associated with autism through a genetic or epigenetic mechanism [19], which could influence the consistency of the findings.

Another main finding of the current study is that excessive GWG might increase the risk of autism among offspring. To date, only a few studies have explored the role of GWG in the risk of autism, and our findings are consistent with those from most previous studies [4–6]. According to Bilder DA et al., GWG was significantly associated with ASD risk (adjusted OR = 1.17, 95% CI: 1.01 to 1.35) [4]. According to Dodds et al., in children with a low genetic susceptibility, pregnancy weight gain greater than 18 kg, which is an excessive weight gain for all BMI classes according to the IOM criteria, is an independent risk factor for autism (OR = 1.21 95% CI: 1.03–1.47) [5]. However, studies on this topic, including the current study, provide only preliminary evidence and do not explain whether excessive GWG is directly or indirectly associated with autism. Therefore, further studies using a prospective design, larger samples and better matching in clinical characteristics remain needed.

In this study, we also conducted a stratification analysis to further explore the potential interactions between BMI and GWG. An excessive GWG might increase the risk of autism among only offspring with overweight/obese mothers and not those with underweight or normal weight mothers. Thus, the interactions between the BMI and GWG may be related to the risk of autism. Connolly et al. reported a similar interaction in their study. These authors found that maternal obesity was associated with only 1.33-fold increased odds of offspring autism, while in obese mothers and mothers with gestational diabetes mellitus (GDM), the odds ratio increased to 2.53 (95% CI: 1.72–3.73) [21]. Furthermore, our result suggests that pre-pregnancy BMI might provide only a susceptible basis. Thus, GWG, which represents the metabolic process and nutritional status during pregnancy, might play a more important role in the etiology or pathophysiology of autism. Leptin, which is a potential biomarker of autism, is an example of a hormone involved in this process [22], and elevated leptin levels predict placental dysfunction [5]. Plasma leptin levels were recently shown to vary in different pre-pregnancy BMI classes, and higher leptin levels may promote greater maternal weight gain during gestation [23]. Additionally, GWG is positively associated with cord blood leptin levels at delivery [24, 25].

This study has some limitations. First, we did not adopt a reliable method to record gestational diseases, such as GDM or gestational hypertension, in the mothers, and these diseases may be important confounding variables related to the risk of autism [26, 27]. Therefore, as mentioned above, we cannot determine whether GWG is directly associated with autism or is only an indication of other metabolic diseases. The second main limitation of this study is that the proportion of obese mothers was small. We did not separately explore the association between BMI and GWG and the risk of autism among obese mothers because of the low statistical power. However, the metabolic or nutritional disturbances may be more obvious in the obese population. Therefore, the association between BMI and GWG and autism in the Chinese population requires further study. Third, the study design has several drawbacks. For example, the subjects were recruited using two different methods, and standardized assessments were lacking, which may limit the validity of the diagnoses and the comparability and representativeness of the population. Another major methodological limitation is the use of parental retrospective self-reports in a case-control study; although the validity of maternal recall of metabolic conditions has gained some support from other studies [28], a false positive result is likely. Hence, more precise and prospective studies are needed.

## Conclusion

This is the largest-scale study investigating this topic in the Chinese Han population. This study adopted a case-control design and provided preliminary evidence about the associations between maternal BMI, GWG and the risk of autism. And the results showed that maternal pre-pregnancy BMI might not be independently associated with the risk of autism, but excessive GWG might increase the risk of autism in offspring of overweight and obese mothers. However, given some limitations of the study, more precise and prospective studies are necessary.

## Additional file

Additional file 1: Table S1. Difference between the Chinese and WHO BMI standards and between the Chinese and IOM recommended GWG standards. **Table S2.** Odds ratios and 95% confidence intervals for the association between the IOM GWG standards and autism using different WHO’s pre-pregnancy BMI classifications. (DOCX 19 kb)

## Abbreviations

ASD: Autism Spectrum Disorder
BMI: Body Mass Index
CI: Confidence interval
GWG: Gestational weight gain
OR: Odds Ratio
TD: Typically developing
