# Supplementary material for: Associations among maternal pre-pregnancy body mass index, gestational weight gain and risk of autism in the Han Chinese population
Source: BMC Psychiatry. 2018 Jan 17;18:11. doi: 10.1186/s12888-018-1593-2 (PMC5773027; doi:10.1186/s12888-018-1593-2)
Supplement: Additional file 1: Table S1. — Difference between the Chinese and WHO BMI standards and between the Chinese and IOM recommended GWG standards. Table S2. Odds ratios and 95% confidence intervals for the association between the IOM GWG standards and autism using different WHO’s pre-pregnancy BMI classifications. (DOCX 19 kb) [file 12888_2018_1593_MOESM1_ESM.docx]

Table S1. Difference between the Chinese and WHO BMI standards and between the Chinese and IOM recommended GWG standards

| Pre-pregnancy BMI | Chinese BMI standards  (kg/m^2^) | WHO BMI standards  (kg/m^2^) | Chinese recommended GWG  (kg) | IOM recommended GWG  (kg) |
| --- | --- | --- | --- | --- |
| Underweight | <18.5 | <18.5 | 15.0-22.0 | 12.5-18.0 |
| Average | 18.5-23.9 | 18.5-24.9 | 13.0-21.0 | 11.5-16.0 |
| Overweight | 24-27.9 | 25.0-29.9 | 10.0-18.0 | 7.0-11.5 |
| Obese | ≥28.0 | ≥30.0 | 9.5-17.0 | 5.0-9.0 |

Table S2. Odds ratios and 95% confidence intervals for the association between the IOM GWG standards and autism using different WHO’s pre-pregnancy BMI classifications

|  | OR | 95% CI | p |
| --- | --- | --- | --- |
|  |  |  |  |
| Underweight ^a^ |  |  |  |
| Inadequate weight gain | 1.443 | 0.831-2.506 | 0.193 |
| Excessive weight gain | 1.122 | 0.658-1.914 | 0.672 |
| Normal ^a^ |  |  |  |
| Inadequate weight gain | 0.761 | 0.569-1.017 | 0.065 |
| Excessive weight gain | 1.026 | 0.790-1.333 | 0.846 |
| Overweight/Obese ^a^ |  |  |  |
| Inadequate weight gain | 0.334 | 0.034-3.279 | 0.347 |
| Excessive weight gain | 4.071 | 1.010-16.641 | 0.048 |

Child’s gender, child age, parental age, and family annual income were used as covariates

^a^ Adequate weight gain group was used as a reference.
